# Periparturient changes in oxidative stress and metabolic parameters in higher-parity Saanen goats with and without kid mortality at birth

**DOI:** 10.1007/s11250-026-05141-3

**Published:** 2026-06-12

**Authors:** Metehan Kutlu, Murat Durmuş

**Affiliations:** 1 Department of Obstetrics and Gynaecology, Faculty of Veterinary Medicine, Necmettin Erbakan University, Ereğli, Konya Türkiye; 2 Department of Animal Science, Faculty of Agriculture, Çukurova University, Adana, Türkiye

**Keywords:** Oxidative stress, Periparturient period, Kid mortality at birth, Dairy goats, Metabolic adaptation

## Abstract

The periparturient period is a critical phase in dairy goats, during which metabolic and oxidative adaptations may influence maternal physiology and offspring viability. This study evaluated longitudinal changes in oxidative stress biomarkers, liver enzyme activities, and metabolic parameters in higher-parity Saanen goats with and without kid mortality at birth. Pregnant goats were followed prospectively and sampled longitudinally from three weeks before kidding to four weeks postpartum. After parturition, goats were grouped according to kid survival status at birth. Kid mortality at birth was defined as the presence of at least one kid that was stillborn or died during parturition before standing and suckling. Goats with kid mortality at birth were assigned to Group 1 (*n* = 6), while matched goats without kid mortality at birth were assigned to Group 2 (*n* = 6). Blood samples were collected at − 21, −14, and − 7 days prepartum, at kidding, and at 24 h, 48 h, 14 days, and 28 days postpartum. Serum total antioxidant status (TAS), total oxidant status (TOS), oxidative stress index (OSI), superoxide dismutase (SOD), paraoxonase-1 (PON-1), malondialdehyde (MDA), catalase (CAT), liver enzymes, and metabolic indicators were analyzed using repeated-measures mixed models. Goats with kid mortality at birth had higher TOS, OSI and SOD concentrations than controls (*P* < 0.05), suggesting increased oxidative burden and antioxidant response. Selected metabolic alterations, including changes in total protein and glucose concentrations, were also observed. Several parameters showed significant time effects and group × time interactions. These findings suggest that kid mortality at birth is associated with systemic oxidative imbalance and selected metabolic changes; however, results should be interpreted as preliminary associations rather than evidence of causality or predictive utility.

## Introduction

The periparturient or transition period represents a critical physiological phase in small ruminants, characterized by profound metabolic, endocrine, and nutritional adaptations required to support parturition and the onset of lactogenesis (Drackley 1999; Radin et al. 2015; Soares et al. 2018). In goats, this period is associated with markedly increased metabolic demands, particularly during the final weeks of gestation when rapid fetal growth occurs. As nutrient intake often fails to meet these elevated requirements, animals frequently experience negative energy balance (NEB) (Mezzetti et al. 2026; Radin et al. 2015). This metabolic challenge promotes the mobilization of body reserves, leading to increased circulating non-esterified fatty acids (NEFA) and enhanced production of reactive oxygen species (ROS) (Chen et al. 2024; Çetin et al. 2021; Eşki et al. 2015).

Excessive ROS generation may disrupt the balance between pro-oxidant and antioxidant systems, resulting in oxidative stress, which has been recognized as an important contributor to impaired reproductive performance (Abd El-Hameed et al. 2018; Celi et al. 2008; Lykkesfeldt and Svendsen 2007; Mutinati et al. 2013; Rizzo et al. 2012; Sies and Cadenas 1985). Although controlled ROS production plays essential roles in fetal development and cellular signaling, excessive oxidative activity can compromise placental function, impair fetal growth, and increase the risk of adverse reproductive outcomes, including stillbirth, embryonic resorption, and reduced neonatal viability (Abd El-Hameed et al. 2018; Al-Gubory et al. 2010; Biondi et al. 2005; Jenkin and Young 2004; Keelan et al. 2003; Mutinati et al. 2013; Rizzo et al. 2012). To counteract oxidative damage, ruminants rely on a complex antioxidant defense system consisting of enzymatic components such as superoxide dismutase (SOD), catalase (CAT), and paraoxonase-1 (PON-1), together with integrated indicators of oxidative balance, including total antioxidant status (TAS) and total oxidant status (TOS) (Celi et al. 2008; El-Sayed et al. 2024; Mohammed et al. 2023).

Kid mortality at birth associated with metabolic and oxidative disturbances represents a major economic and welfare concern in goat production, resulting in reduced productivity, increased veterinary costs, and direct offspring loss (Jacobson et al. 2020; Uztimür and Ünal 2024). Despite its biological and economic importance, the relationship between oxidative status and reproductive performance in goats remains insufficiently investigated compared with other livestock species, representing a significant gap in the current literature (Abdel-Ghani et al. 2016; Karapehlivan et al. 2013; Soares et al. 2018).

Furthermore, current research on periparturient oxidative stress in goats has primarily focused on transition physiology or specific metabolic disorders, such as pregnancy toxemia, while its direct association with kid mortality at birth has received limited attention (Radin et al. 2015; Santarosa et al. 2021; Uztimür and Ünal 2024). In particular, studies simultaneously evaluating systemic oxidative stress biomarkers alongside metabolic and hepatic indicators, such as aspartate aminotransferase (AST), alanine aminotransferase (ALT), and gamma-glutamyl transferase (GGT), in relation to kid mortality at birth are scarce. Such an integrated approach is essential for elucidating the complex pathophysiological mechanisms underlying kid mortality and for identifying potential biomarkers that may predict adverse reproductive outcomes.

Therefore, this study aimed to evaluate oxidative stress markers and metabolic parameters in higher-parity Saanen goats with and without kid mortality at birth. We hypothesized that higher-parity goats experiencing kid mortality at birth would exhibit increased oxidative stress and altered metabolic parameters during the periparturient period compared with goats without kid mortality at birth.

## Materials and methods

The experimental procedures were approved by the Animal Experiments Local Ethics Committee of Cukurova University (Adana, Türkiye) (22.10.2024–10-4).

### Location and farm

The experiment was carried out at the Research Farm of the Faculty of Agriculture, Çukurova University, Adana, Türkiye (37°04′43.3″ N, 35°37′22.6″ E; altitude: 23 m above sea level). Initially, 50 clinically healthy Saanen goats in their 4th–9th lactation, with body weights ranging from 50 to 65 kg, were included in the study.

### Synchronization, estrus detection and matings

All goats were treated with intravaginal sponges containing 60 mg medroxyprogesterone acetate for 13 days (Day 0; Esponjavet^®^, Hipra, Spain). At sponge withdrawal (Day 13), 500 IU equine chorionic gonadotropin (eCG; Oviser^®^, Hipra, Spain) was administered intramuscularly. Estrus detection was initiated 12 h after sponge removal using teaser bucks. Goats exhibiting estrus were naturally mated by hand mating at a buck-to-goat ratio of 1:7. Pregnancy diagnosis was performed 40–42 days after mating by transabdominal ultrasonography using a 3.5-MHz probe (EUB-405, Hitachi, Japan).

### Design of the experiment, animals and diet

Pregnant goats were followed prospectively and sampled longitudinally at predefined time points from three weeks before kidding to four weeks postpartum. Initially, 50 clinically healthy pregnant goats were monitored during the study period. After parturition, goats were evaluated according to kid survival status at birth and the predefined eligibility criteria. In the present study, kid mortality at birth was defined as the presence of at least one kid that was stillborn or died during parturition before standing and suckling. Kid deaths occurring after the immediate birth period, including deaths within the first 24 h postpartum, were not included in this endpoint. All goats with at least one kid meeting this definition and fulfilling the inclusion criteria were assigned to Group 1 (kid mortality at birth; *n* = 6). Among goats whose kids were alive at birth, control goats were selected based on the closest similarity to Group 1 goats with respect to litter size and lactation number, in order to reduce potential confounding related to reproductive performance and metabolic status. Accordingly, six matched goats were assigned to Group 2 (no kid mortality at birth; *n* = 6). Goats that did not meet the definition of kid mortality at birth and were not selected as matched controls were not included in the final analysis. Therefore, the final analytical cohort consisted of 12 multiparous goats (lactation number range: 4–8). Descriptive characteristics of the goats are presented in Table 1.

**Table 1:** Descriptive characteristics of the goats used in the study

Animal Number	Healthy Kid	Dead Kid	Total Kid	Lactation Number
*Group 1 (Kid mortality at birth)*				
Goat 1	3	1	4	6
Goat 2	3	1	4	6
Goat 3	2	1	3	6
Goat 4	2	1	3	7
Goat 5	2	1	3	4
Goat 6	1	1	2	8
*Group 2 (Control)*				
Goat 7	3	-	3	6
Goat 8	3	-	3	6
Goat 9	3	-	3	6
Goat 10	3	-	3	6
Goat 11	3	-	3	4
Goat 12	2	-	2	6

All goats were maintained under the same management conditions throughout the study period. Animals were housed in the same farm environment and received the same feeding program, water access, and routine care. No major changes in housing, feeding, or general management practices occurred during the monitoring period, and no apparent management-related problems were observed. Therefore, management conditions were considered standardized across animals; however, the potential influence of unmeasured individual-level factors cannot be completely excluded. During the kidding period, animals were monitored routinely by farm personnel, and no major dystocia, infectious disease outbreak, abrupt dietary change, or management-related event was recorded.

Diets were formulated to meet NRC (2007) requirements. All goats were offered a total mixed ration (TMR) at 2,750 g per head per day; however, this value refers to the amount of feed offered per animal and does not represent measured individual feed intake. Actual individual feed consumption and dry matter intake could not be determined under the present farm conditions. During the prepartum period, the forage-to-concentrate ratio was formulated at 50:50. The forage component consisted of chopped alfalfa hay (30%) and chopped wheat straw (70%), while the concentrate fraction comprised a commercial dairy concentrate containing 20% crude protein. During the postpartum period, the forage-to-concentrate ratio was adjusted to 40:60, and the same commercial dairy concentrate was used. The forage portion of the TMR consisted of chopped alfalfa hay (75%) and chopped wheat straw (25%). Detailed ration compositions are presented in Tables 2 and 3.

**Table 2:** Composition of the concentrate feed used in the ration

Feed ingredient composition	%
Corn	41.4
Barley	11.3
Vibratol	3.0
Soybean meal (48% crude protein)	17.4
Wheat bran	7.5
Corn-based dried distillers grains with solubles (DDGS)	15.1
Fractionated fat	1.5
Salt	0.8
Vitamin–mineral premix¹	0.1
Sodium bicarbonate	0.8
Calcium carbonate	1.1

¹Vitamin–mineral premix per kg contains: 15,000,000 IU vitamin A, 3,000,000 IU vitamin D₃, 30,000 mg vitamin E, 150,000 mg niacin, 10,000 mg Cu, 800 mg I, 150 mg Co, 150 mg Se, 50,000 mg Mn, 50,000 mg Fe, 50,000 mg Zn, 6,800 mg organic Mn, 1,400 mg organic Cu, 6,800 mg organic Zn, 6,800 mg organic Fe, and 50 mg organic Se

**Table 3:** Nutrient composition of the TMRs used (on a dry matter basis)

Nutrients	Prepartum	Postpartum
Crude protein, %	14.66	17.86
Rumen undegradable protein, % of crude protein	5.93	8.09
Rumen degradable protein, % of crude protein	8.73	9.78
NDF, %	53.37	41.58
ADF, %	30.40	23.28
Ether extract, %	2.88	3.34
Ash, %	8.71	8.45
Net energy for lactation (NEl) (Mcal/kg)	1.26	1.34

### Blood collection and measurements

Blood samples were collected from all goats at − 21, −14, and − 7 days prepartum; at kidding (0 h); and at 24 h, 48 h, 14 days, and 28 days postpartum. Serum samples were analyzed for oxidative stress biomarkers, including total antioxidant status (TAS), total oxidant status (TOS), superoxide dismutase (SOD), paraoxonase-1 (PON-1), malondialdehyde (MDA), and catalase (CAT); liver enzyme activities, including aspartate aminotransferase (AST), alanine aminotransferase (ALT), and gamma-glutamyl transferase (GGT); and metabolic parameters (albumin, total cholesterol, glucose, total protein, triglycerides, and urea). Blood samples were obtained from the jugular vein into sterile gel vacuum tubes and centrifuged at 4,000 rpm for 10 min (Universal 320R, Hettich, Germany). Serum was separated, aliquoted, and stored at − 20 °C until analysis. Serum biochemical parameters (AST, ALT, GGT, albumin, total cholesterol, glucose, total protein, triglycerides, urea, SOD, and CAT) were determined using commercial assay kits (Otto Scientific, Türkiye). TAS, TOS, and PON-1 levels were measured using commercial kits (Rel Assay Diagnostics, Türkiye) with an automated biochemical analyzer (BS-400, Mindray, China). Serum MDA concentrations were measured colorimetrically using a commercial assay kit (Otto Scientific, Türkiye) according to the manufacturer’s instructions. The oxidative stress index (OSI) was calculated as TOS/TAS × 100 (Abuelo et al. 2013; Giorgio et al. 2020). All assays were performed according to manufacturer instructions and quality control procedures. Samples were analyzed in single runs.

### Statistical analysis

All data were initially organized in Microsoft Excel (version 2013; Microsoft Corporation, USA) and subsequently analyzed using the GLIMMIX procedure of SAS 9.4 software (SAS Institute Inc., Cary, NC, USA). Periparturient-period measurements were analyzed using repeated-measures mixed models, with time specified as the repeated factor and animal included as a random effect (subject = animal). The statistical model included group, time, and their interaction (group × time) as fixed effects, while litter size and lactation number were included as covariates. The optimal covariance structure for repeated measures was selected based on the lowest Akaike Information Criterion (AIC), and a heterogeneous first-order autoregressive structure [ARH(1)] was applied (Çetin and Bek 2019). Model residuals were assessed for normality using the Shapiro–Wilk test. Pairwise comparisons were adjusted using the Tukey–Kramer method. Results are presented as least squares means ± standard error (LSM ± SE), and statistical significance was declared at *P* < 0.05.

An a priori sample size estimation was not performed due to the exploratory nature of the study and the limited availability of animals experiencing kid mortality within the herd. Therefore, a post hoc power analysis was conducted to evaluate the adequacy of the sample size for detecting between-group differences in the primary oxidative stress outcomes. Power calculations were performed using G*Power software (version 3.1; Heinrich-Heine-Universität Düsseldorf, Germany). Because the main analyses were based on repeated-measures mixed models, a conservative approach was applied by estimating effect sizes from the observed least-squares means (LSM) and standard errors (SE) of the principal biomarkers. Standard deviations were derived from SE values (SD = SE × √n; *n* = 6 per group), and Cohen’s d was calculated assuming independent two-group comparisons (two-tailed test, α = 0.05, allocation ratio 1:1). Based on the observed differences between groups, the estimated effect size for oxidative stress index (OSI; 0.60 ± 0.02 vs. 0.48 ± 0.02) was d = 2.45, corresponding to an achieved statistical power of 0.968. For total oxidant status (TOS; 7.80 ± 0.30 vs. 6.19 ± 0.25), the estimated effect size was d = 2.38 with an achieved power of 0.959. For superoxide dismutase (SOD; 285.3 ± 3.0 vs. 273.9 ± 2.5), the effect size was d = 1.69 and the achieved power was 0.749. These results indicate that the current sample size provided adequate power to detect large differences in primary oxidative stress markers, whereas smaller or moderate effects may not have been detected. For future prospective studies aiming to detect moderate effect sizes (d = 0.80) with 80% statistical power at α = 0.05, a minimum sample size of approximately 26 animals per group would be required.

## Results

**Fig. 1:**
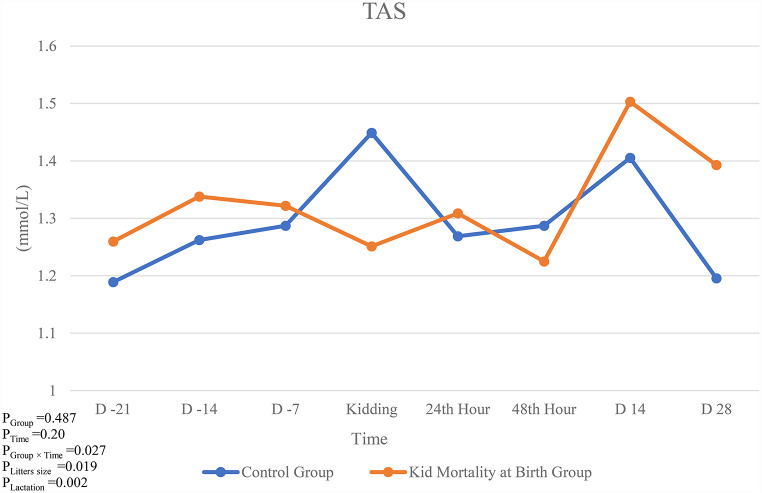
TAS concentrations in goats during the peripartum period

**Fig. 2:**
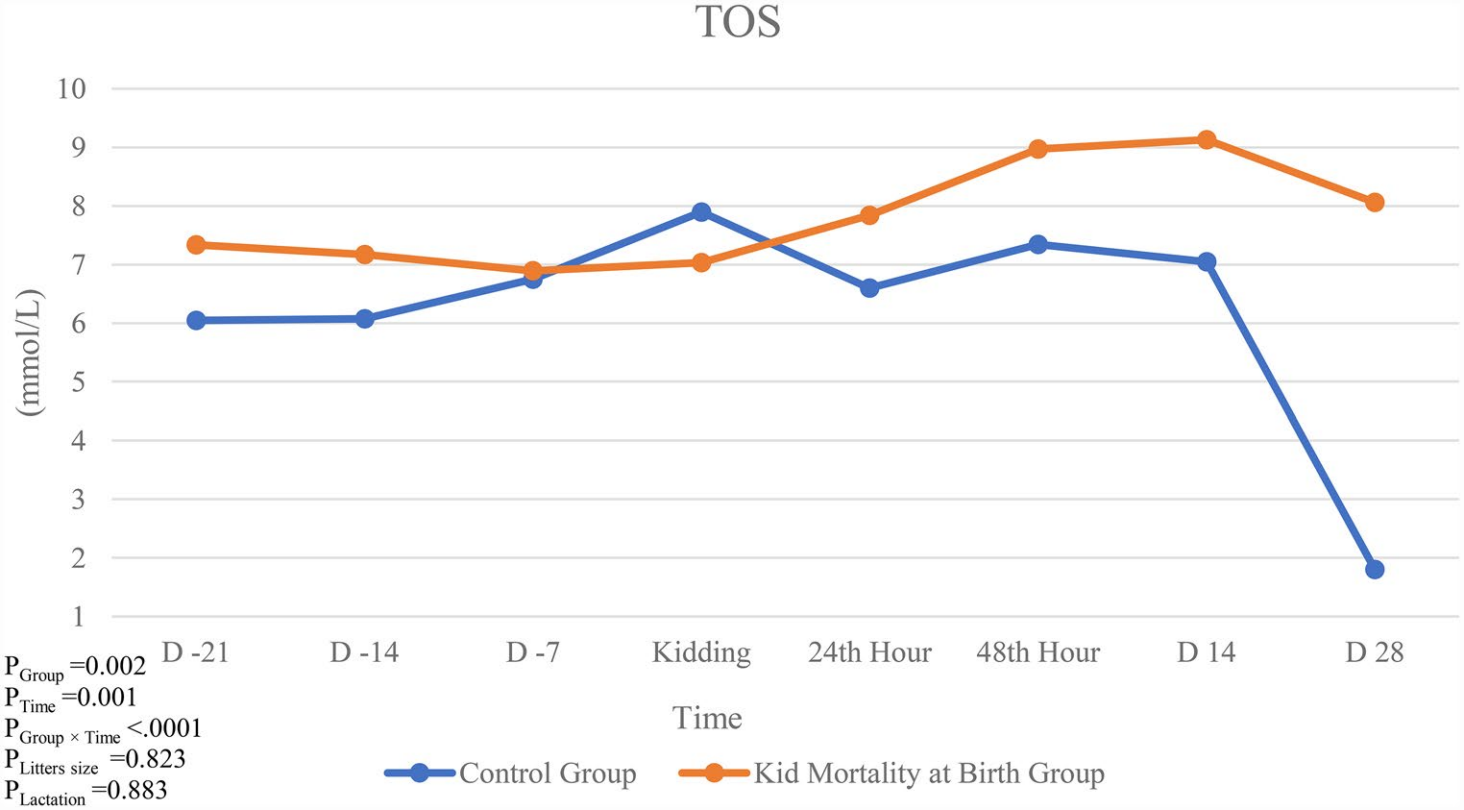
TOS concentrations in goats during the peripartum period

**Fig. 3:**
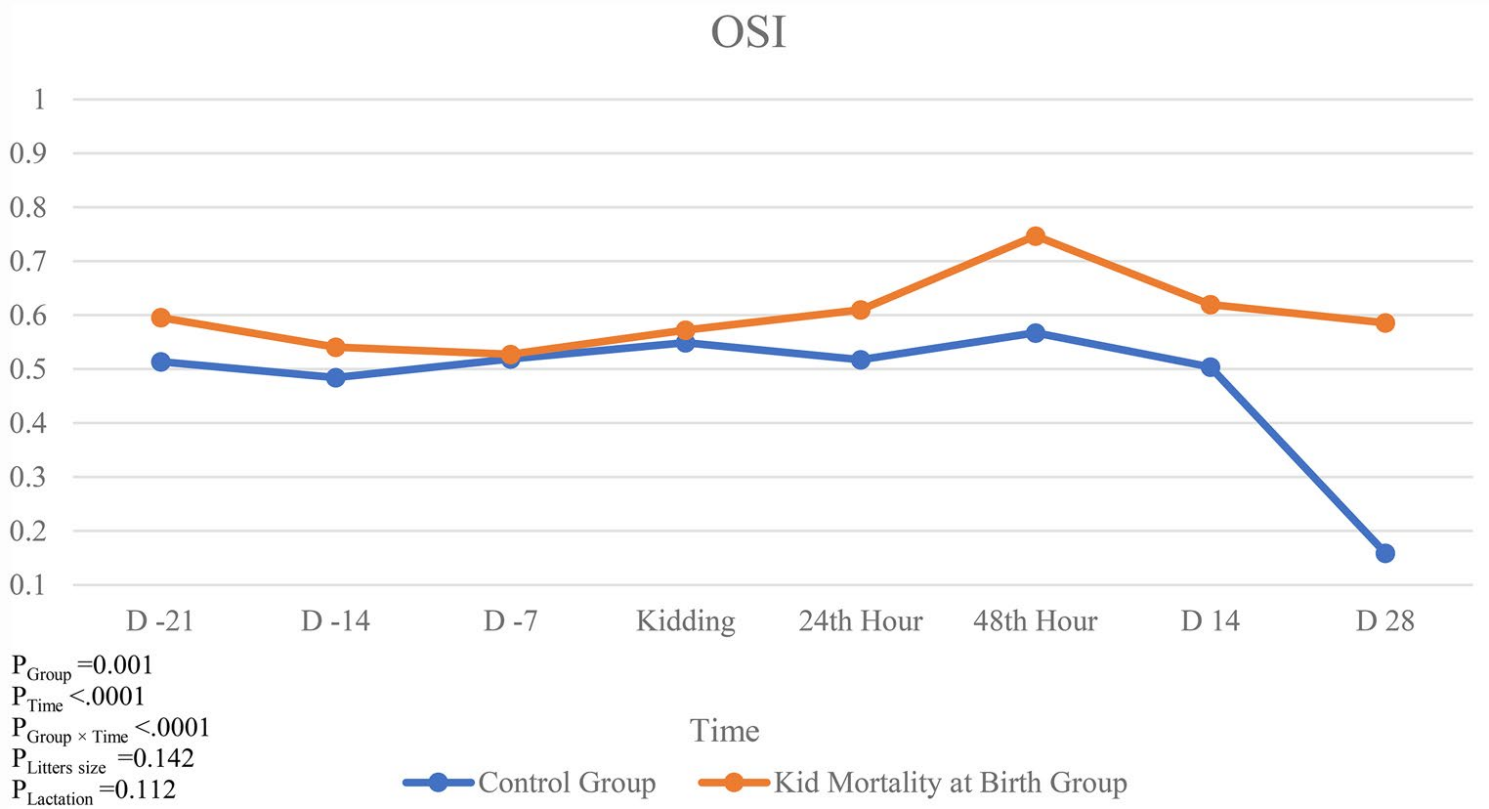
OSI levels in goats during the peripartum period

**Fig. 4:**
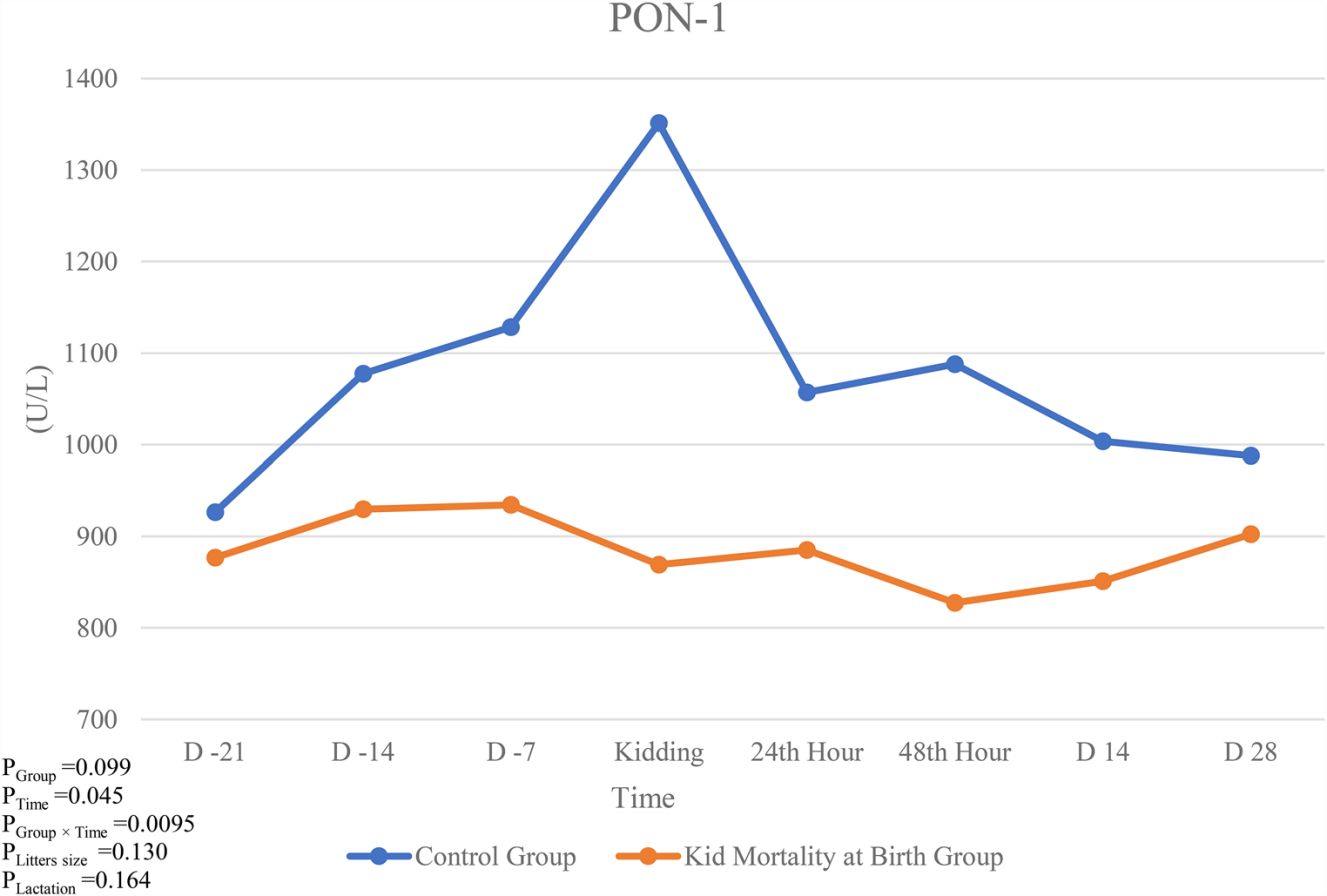
PON-1 concentrations in goats during the peripartum period

**Fig. 5:**
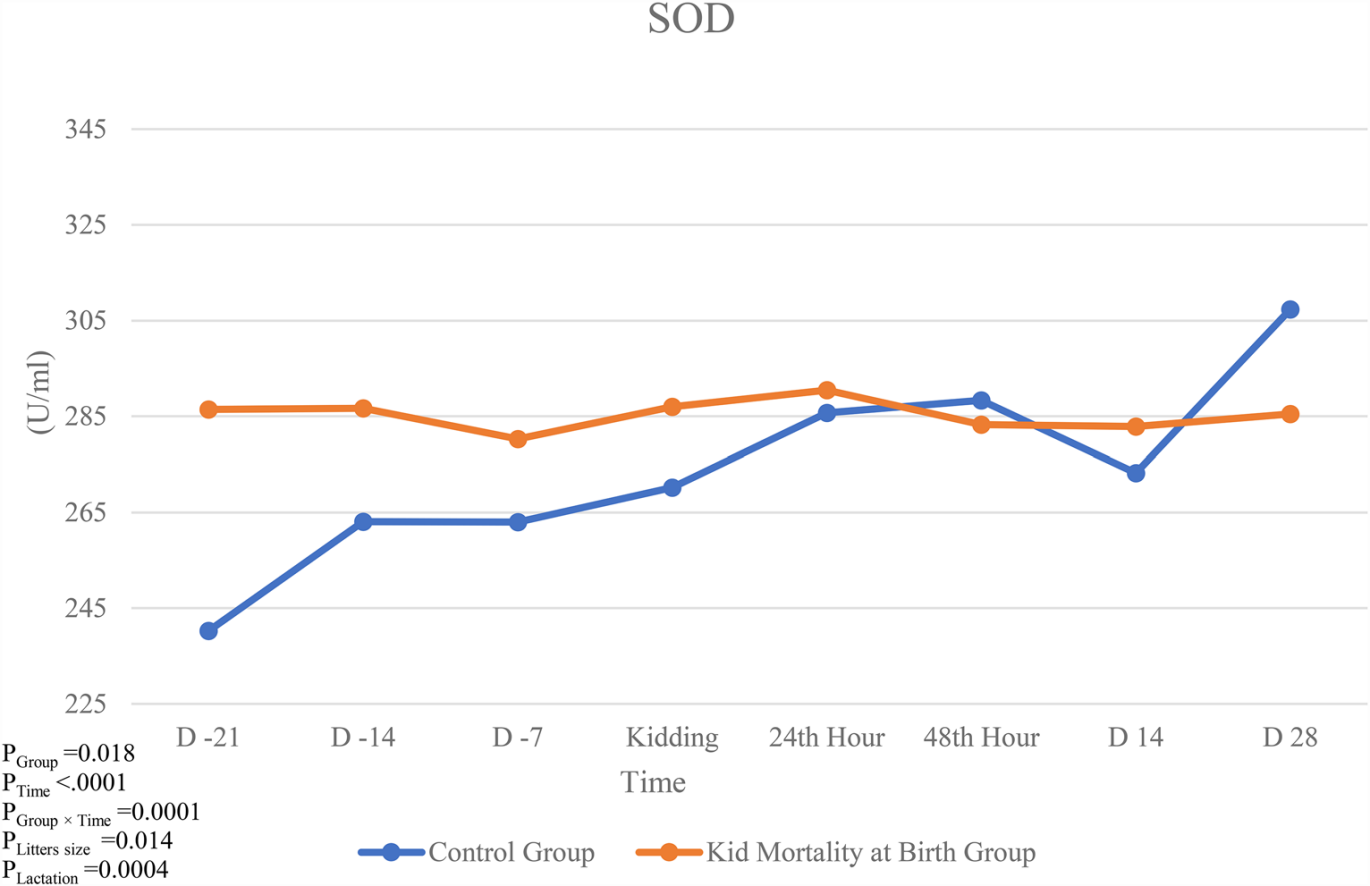
SOD concentrations in goats during the peripartum period

**Fig. 6:**
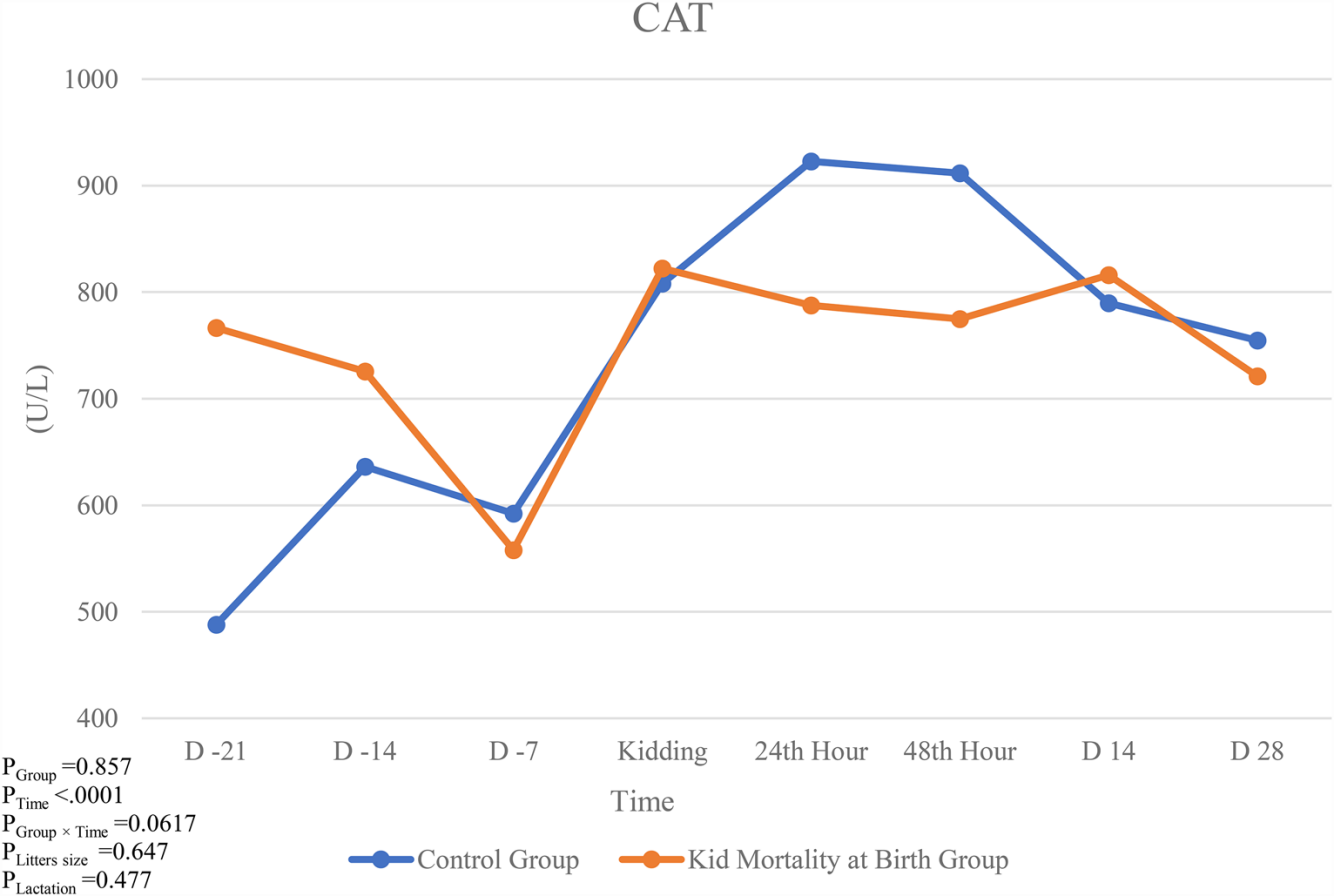
CAT concentrations in goats during the peripartum period

**Fig. 7:**
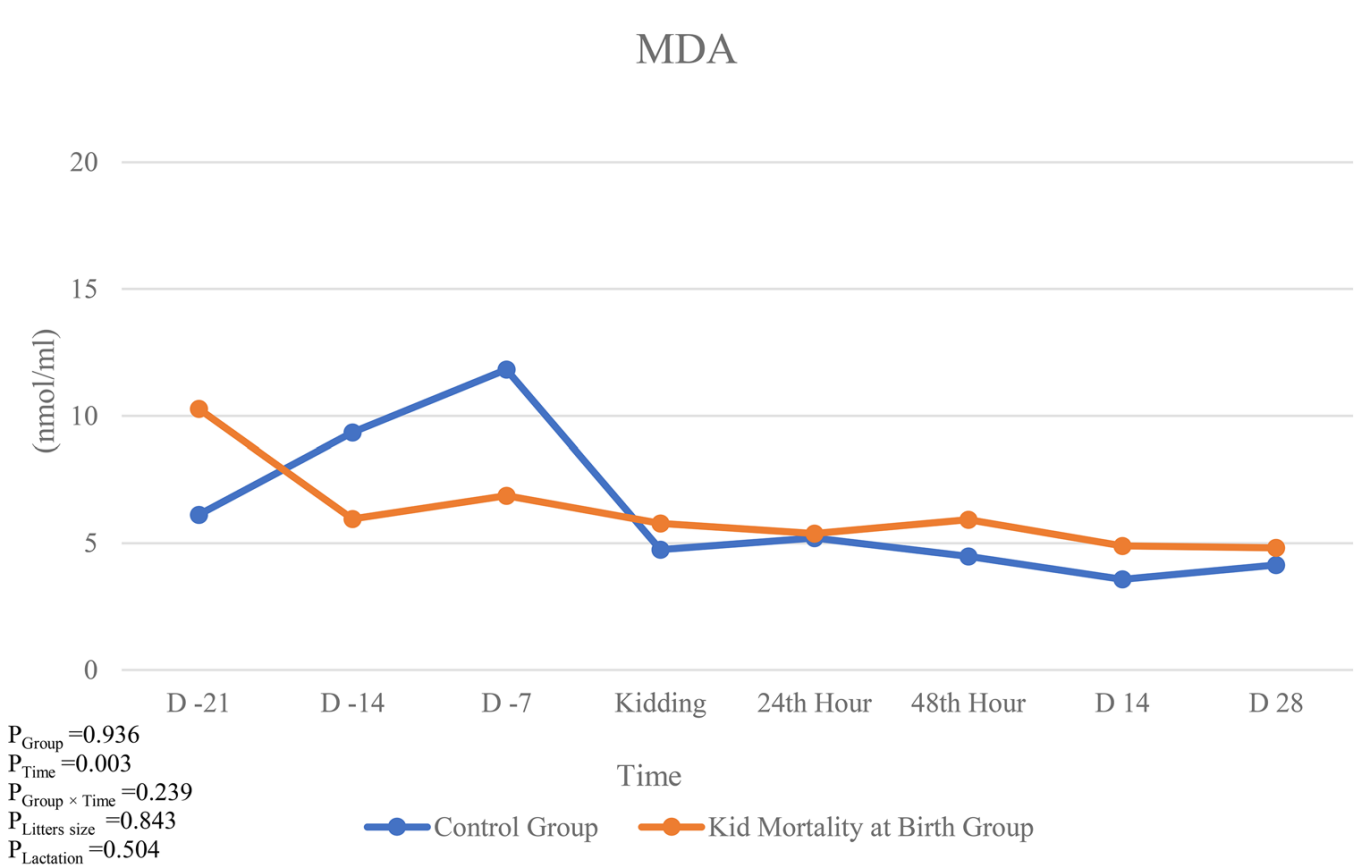
MDA concentrations in goats during the peripartum period

Serum oxidative stress biomarker concentrations are presented in Table 4. TOS, OSI, and SOD concentrations were significantly higher in the kid mortality at birth group than in the control group (*P* = 0.002, *P* = 0.001, and *P* = 0.018, respectively). No significant group effects were observed for TAS, CAT, PON-1 and MDA concentrations (*P* > 0.05). Time significantly affected all oxidative stress parameters (*P* < 0.05). Significant group × time interactions were observed for TAS, TOS, OSI, SOD, and PON-1 (*P* < 0.05), whereas CAT and MDA showed no interaction effects (Figs. 1, 2, 3, 4, 5, 6 and 7).

**Table 4:** Serum oxidative stress marker concentrations

LSM ± SE	Group 1 (Kid mortality at birth)	Group 2 (Control)	*P*				
			Group	Time	Group × Time	Litter Size	Lactation Number
TAS (mmol/L)	1.33 ± 0.03	1.29 ± 0.03	0.487	0.020	0.027	0.019	0.002
TOS (mmol/L)	7.80 ± 0.30	6.19 ± 0.25	0.002	0.001	< 0.0001	0.823	0.883
OSI	0.60 ± 0.02	0.48 ± 0.02	0.001	< 0.0001	< 0.0001	0.142	0.112
SOD (U/ml)	285.3 ± 3.0	273.9 ± 2.5	0.018	< 0.0001	0.0001	0.014	0.0004
CAT (U/L)	746.6 ± 31.7	738.0 ± 27.4	0.857	< 0.0001	0.0617	0.647	0.477
PON-1 (U/L)	884.6 ± 81.1	1077.5 ± 72.5	0.099	0.045	0.0095	0.130	0.164
MDA (nmol/ml)	6.24 ± 0.52	6.18 ± 0.46	0.936	0.003	0.239	0.843	0.504

**Fig. 8:**
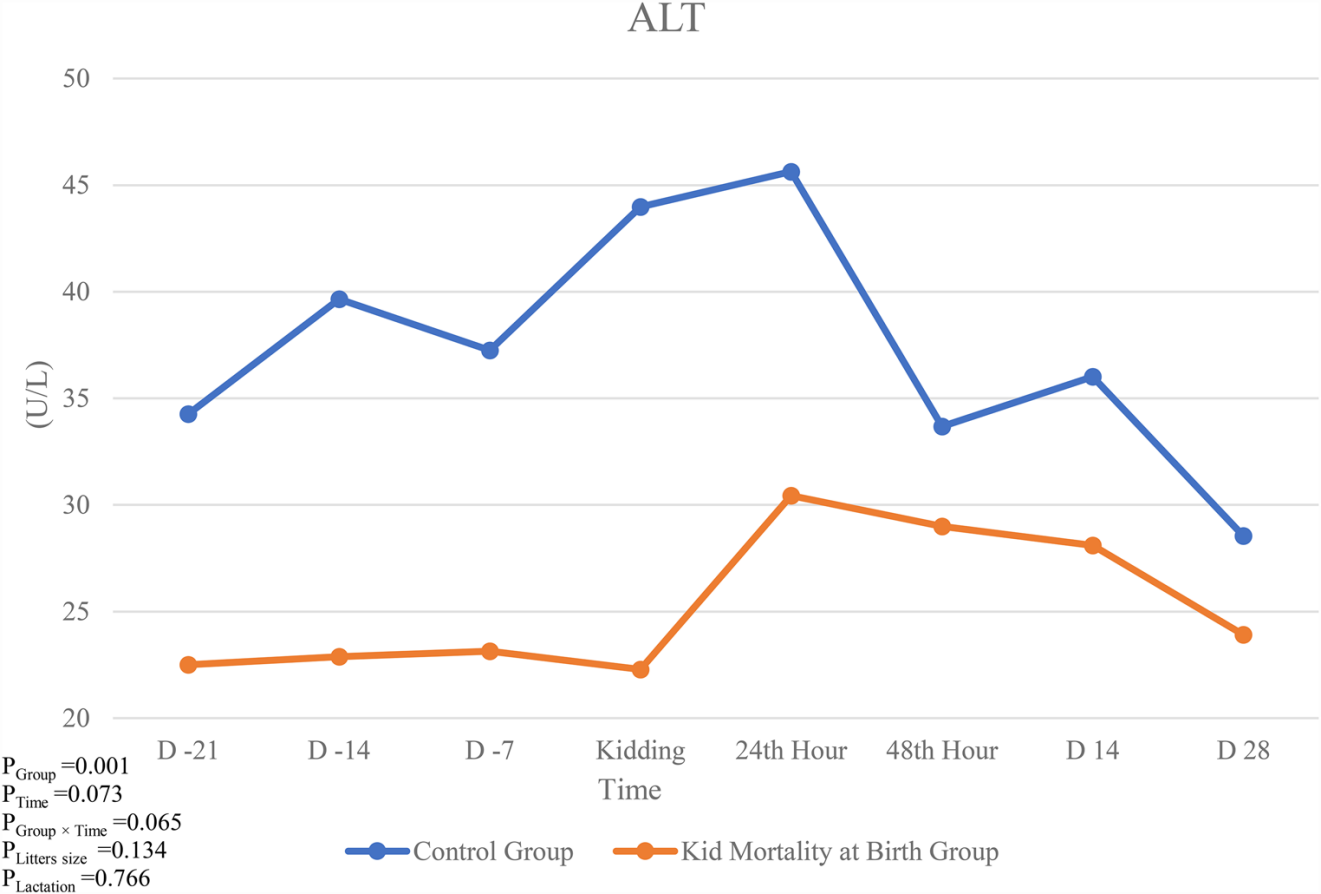
ALT concentrations in goats during the peripartum period

**Fig. 9:**
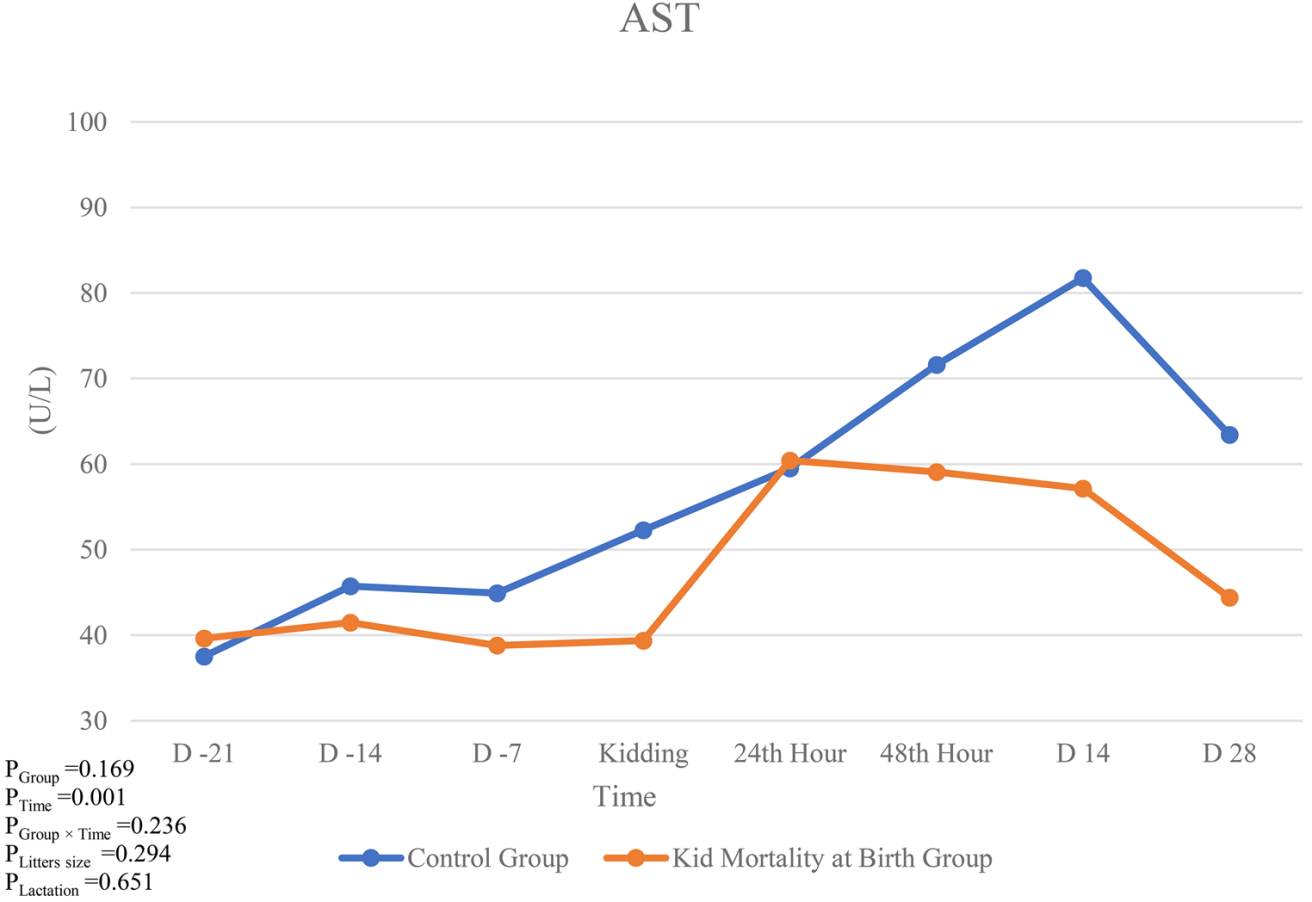
AST concentrations in goats during the peripartum period

**Fig. 10:**
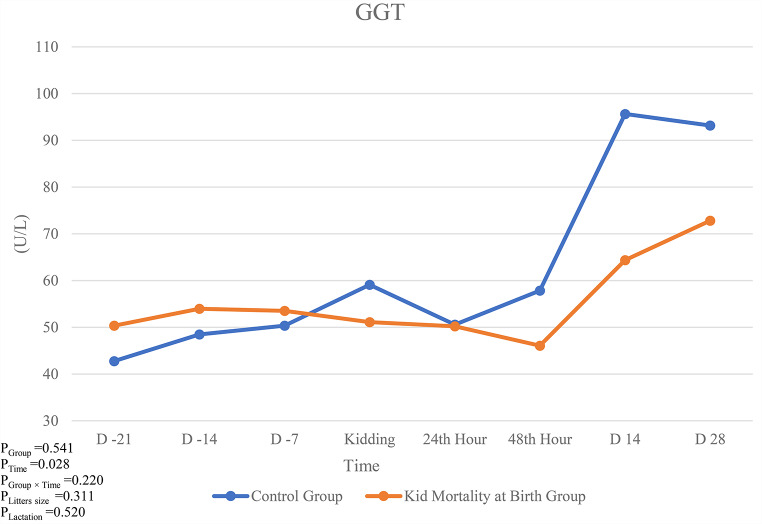
GGT concentrations in goats during the peripartum period

**Fig. 11:**
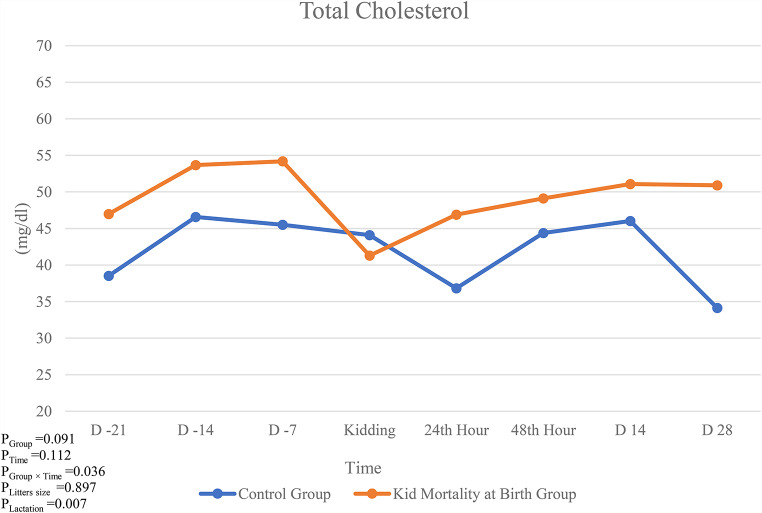
Total cholesterol concentrations in goats during the peripartum period

**Fig. 12:**
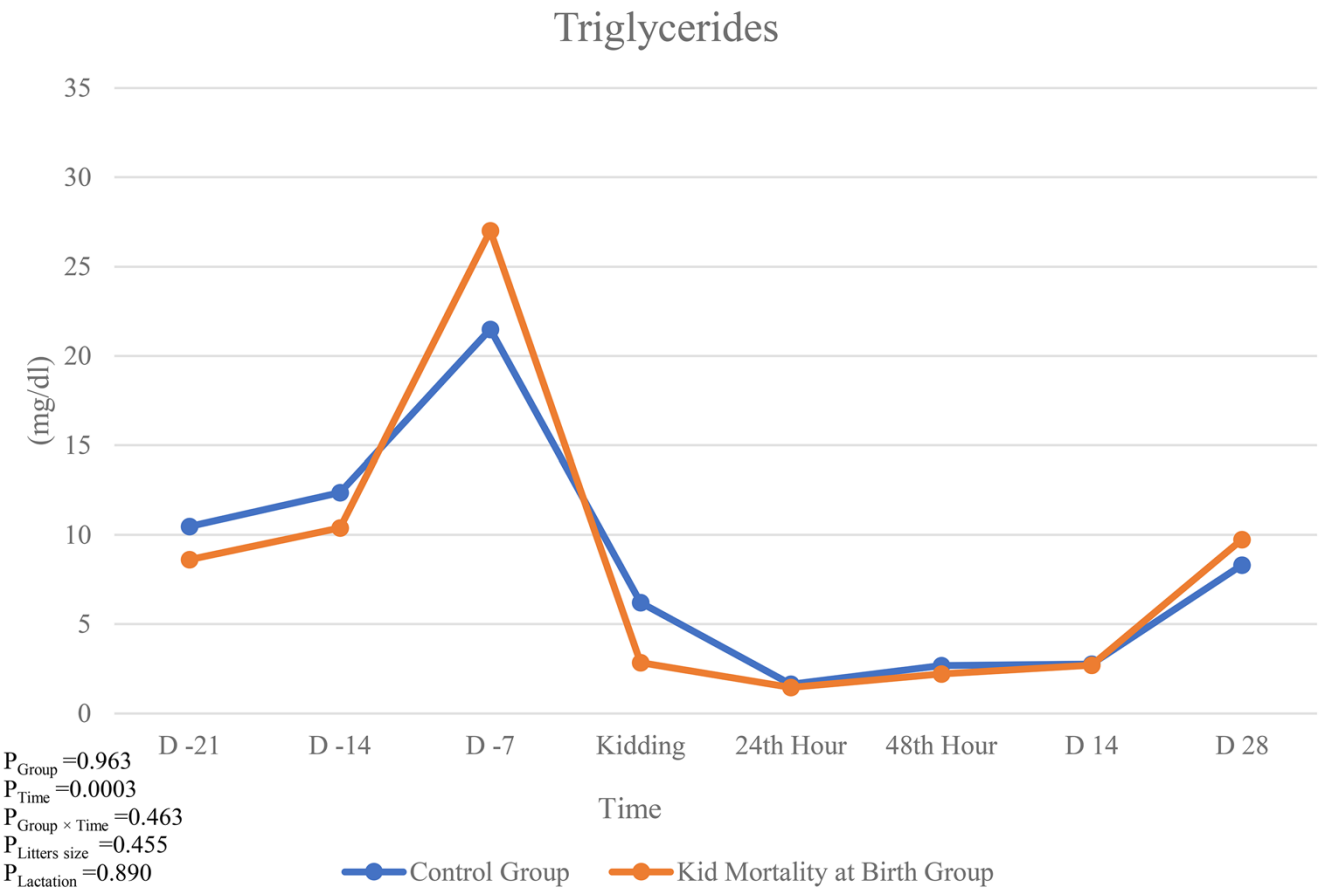
Triglycerides concentrations in goats during the peripartum period

**Fig. 13:**
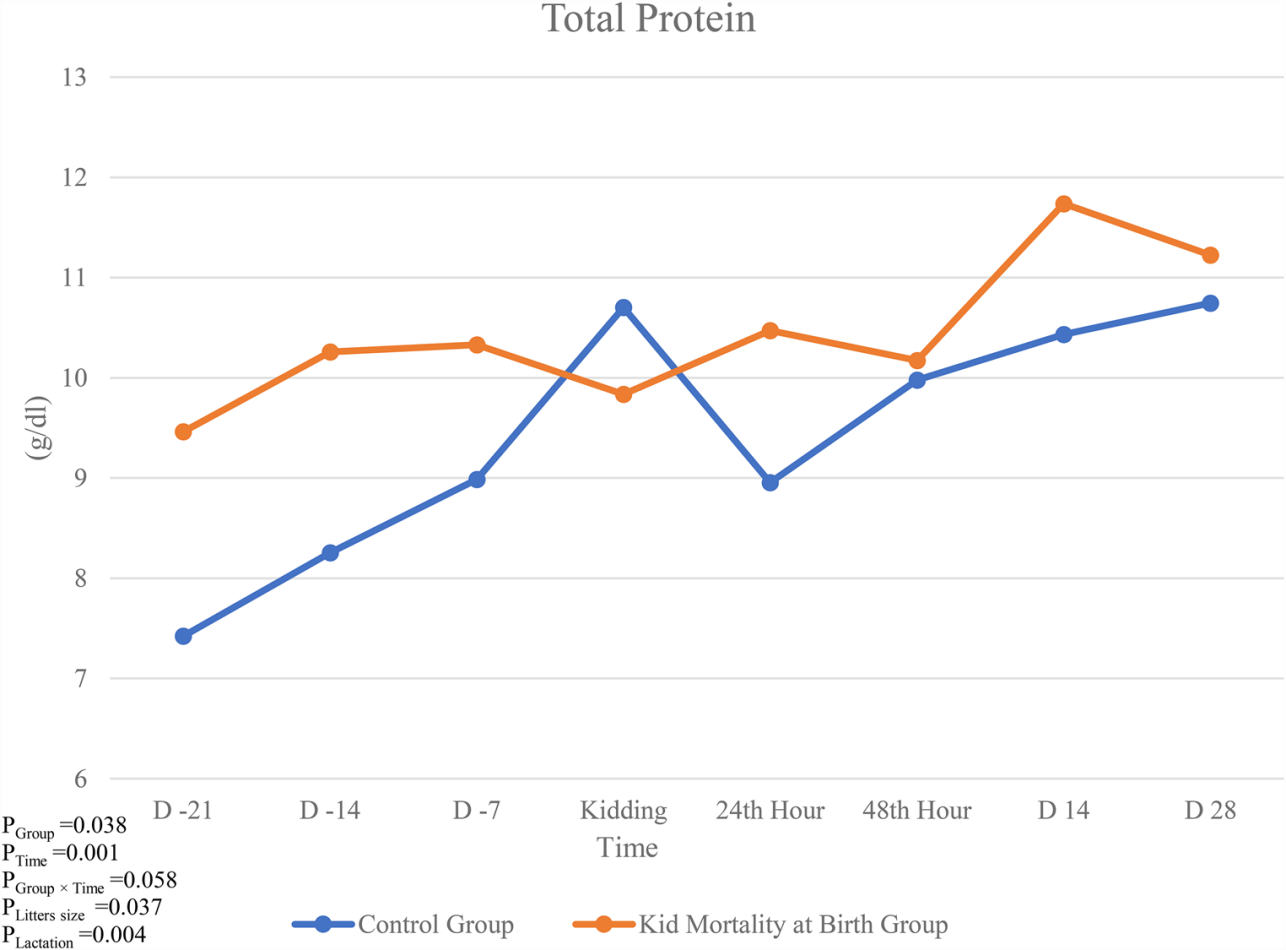
Total protein concentrations in goats during the peripartum period

**Fig. 14:**
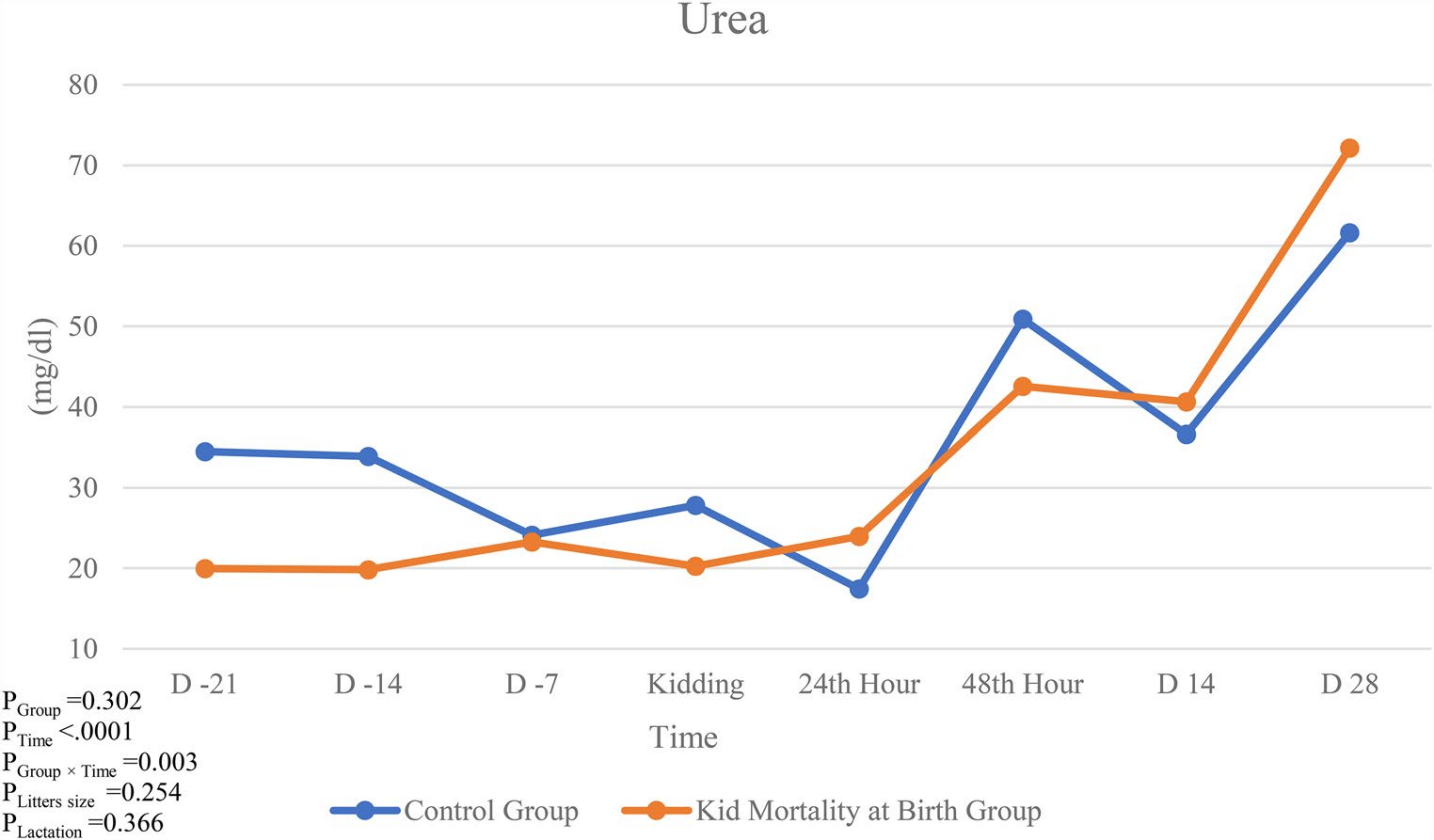
Urea concentrations in goats during the peripartum period

**Fig. 15:**
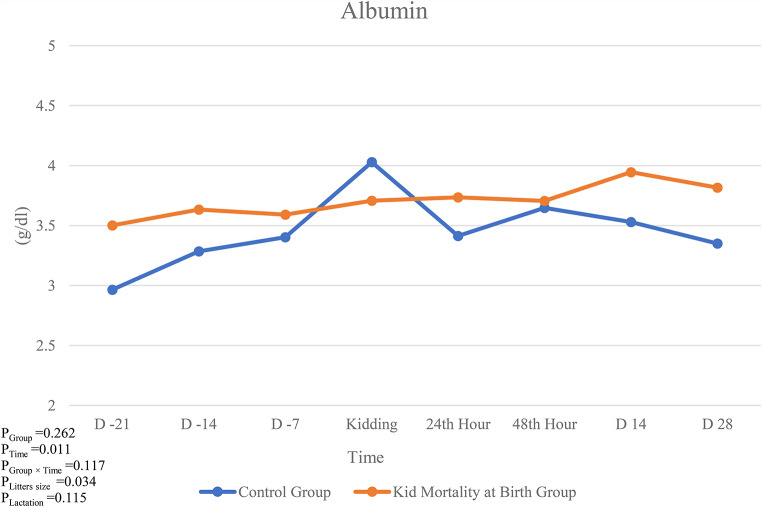
Albumin concentrations in goats during the peripartum period

**Fig. 16:**
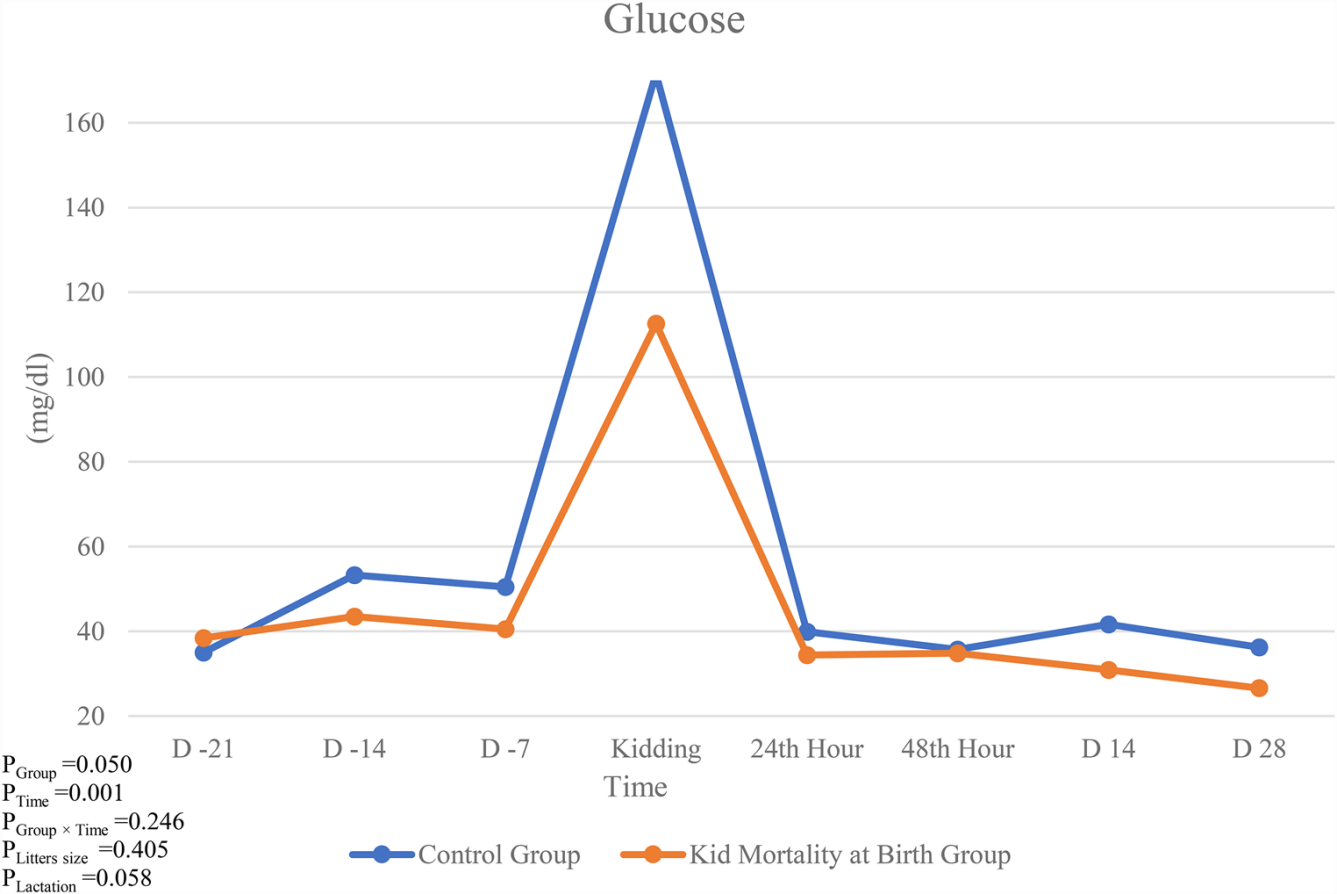
Glucose concentrations in goats during the peripartum period

Serum biochemical parameters are summarized in Table 5 (Figs. 8, 9, 10, 11, 12, 13, 14, 15 and 16). ALT activity was significantly lower in the kid mortality at birth group than in the control group (*P* = 0.001), whereas total protein concentrations were significantly higher (*P* = 0.038). Glucose concentrations tended to differ between groups (*P* = 0.050). No significant group effects were detected for AST, GGT, total cholesterol, triglycerides, urea, or albumin (*P* > 0.05).

**Table 5:** Serum biochemical marker concentrations

LSM ± SE	Group 1 (Kid mortality at birth)	Group 2 (Control)	*P*				
			Group	Time	Group × Time	Litter Size	Lactation Number
ALT (U/L)	25.3 ± 2.01	37.4 ± 1.72	0.001	0.073	0.065	0.134	0.766
AST (U/L)	47.5 ± 4.6	57.1 ± 4.0	0.169	0.001	0.236	0.294	0.651
GGT (U/L)	55.3 ± 7.5	62.3 ± 6.50	0.541	0.028	0.220	0.311	0.520
T. Cholesterol (mg/dl)	49.3 ± 2.7	42.0 ± 2.32	0.091	0.112	0.036	0.897	0.007
Triglycerides (mg/dl)	8.12 ± 1.89	8.24 ± 1.66	0.963	0.0003	0.463	0.455	0.890
Total Protein (g/dl)	10.4 ± 0.3	9.44 ± 0.26	0.038	0.001	0.058	0.037	0.004
Urea (mg/dl)	32.8 ± 2.0	35.9 ± 1.73	0.302	< 0.0001	0.003	0.254	0.366
Albumin (g/dl)	3.71 ± 0.14	3.45 ± 0.12	0.262	0.011	0.117	0.034	0.115
Glucose (mg/dl)	45.2 ± 4.4	58.0 ± 3.91	0.050	0.001	0.246	0.405	0.058

Time significantly influenced AST, GGT, triglycerides, total protein, urea, albumin, and glucose concentrations (*P* < 0.05), whereas ALT and total cholesterol were not affected by time. Significant group × time interactions were detected for total cholesterol and urea (*P* < 0.05), while no interaction effects were observed for the remaining biochemical parameters.

## Discussion

This study provides preliminary exploratory data suggesting that systemic oxidative imbalance during the periparturient period may be associated with kid mortality at birth in dairy goats. By evaluating temporal changes in oxidative stress biomarkers together with metabolic and hepatic indicators, the present study contributes to the limited available information on maternal biochemical alterations related to kid survival in goats. Goats experiencing kid mortality at birth showed a higher oxidative burden, as reflected by increased TOS and OSI values, together with changes in antioxidant enzyme activity and metabolic parameters. These findings suggest that disruption of redox homeostasis during the periparturient period may be associated with adverse kid survival outcomes. However, given the small sample size and post-parturition group allocation, these results should be interpreted cautiously as exploratory associations rather than evidence of causality or prediction. Further studies with larger prospective cohorts are required to confirm these observations and clarify their biological relevance.

The increased TOS and OSI concentrations observed in goats with kid mortality at birth suggest a shift toward pro-oxidant status and altered redox balance. The periparturient period is characterized by intense metabolic demands associated with rapid fetal growth, parturition, and onset of lactation, which promote lipolysis, mitochondrial activity, and increased production of reactive oxygen species (ROS) (Bernabucci et al. 2005; Celi and Gabai 2015; Sordillo and Aitken 2009). In dairy goats, this transition period is accompanied by marked changes in metabolic and oxidative status, reflecting the physiological challenges of maintaining energy balance and redox homeostasis (Celi et al. 2008; Radin et al. 2015). Previous studies have demonstrated dynamic alterations in oxidant and antioxidant parameters across different physiological stages of pregnancy and lactation in goats, indicating increased susceptibility to oxidative imbalance during periods of elevated metabolic demand (Çetin et al. 2021; Karapehlivan et al. 2013). Increased ROS generation during this period has been linked to negative energy balance, immune dysfunction, and impaired reproductive performance in ruminants (Castillo et al. 2006; Celi 2010). Although the present study did not directly evaluate placental function, inflammatory mediators, neonatal pathology, or endocrine regulation, several possible mechanisms may help explain the observed association between systemic oxidative imbalance and kid mortality at birth. These may include alterations in mitochondrial function, placental development, endocrine regulation, and inflammatory processes associated with parturition. Reactive oxygen species are known to play important regulatory roles in female reproductive physiology, including follicular development, steroidogenesis, implantation, and placental function; however, excessive ROS production may disrupt these processes and impair reproductive outcomes (Rizzo et al. 2012). Oxidative imbalance has also been associated with inflammatory pathways involved in the initiation of parturition, where cytokines and prostaglandins regulate uterine activity and fetal membrane function (Keelan et al. 2003). Moreover, complex endocrine and physiological mechanisms controlling parturition are highly sensitive to metabolic and oxidative disturbances, which may compromise fetal survival and neonatal viability (Jenkin and Young 2004). Therefore, these mechanisms should be interpreted as biologically plausible explanations rather than directly tested pathways in the present study. Further studies including placental evaluation, inflammatory markers, neonatal pathological assessment, and endocrine measurements are required to clarify the mechanisms linking oxidative imbalance with kid mortality at birth.

Interestingly, SOD activity was significantly higher in goats experiencing kid mortality at birth. This increase likely represents a compensatory response to excessive superoxide radical production rather than improved antioxidant protection. Superoxide dismutase constitutes the first enzymatic barrier against oxidative damage, and increased activity generally reflects activation of endogenous defense systems under oxidative challenge (Celi and Gabai 2015; Halliwell and Gutteridge 2015; Lykkesfeldt and Svendsen 2007). Similar compensatory increases in antioxidant enzyme activity have been reported in small ruminants exposed to metabolic stress or pregnancy-related oxidative imbalance (Abd El-Hameed et al. 2018; Di Trana et al. 2006; Mutinati et al. 2013; Santarosa et al. 2021). In particular, previous studies in ewes and goats have demonstrated significant alterations in antioxidant enzyme activities during pregnancy and under conditions of increased metabolic demand, suggesting activation of endogenous defense mechanisms in response to oxidative challenge (Mutinati et al. 2013; Santarosa et al. 2021). Likewise, changes in oxidant and antioxidant indices in pregnant ewes and goats have been associated with variations in reproductive status and fetal number, further supporting the role of oxidative stress in reproductive physiology (Abd El-Hameed et al. 2018). In the present study, the absence of significant differences in TAS, CAT, and MDA concentrations between groups suggests that total antioxidant capacity and lipid peroxidation markers may not fully capture early or localized oxidative damage. Alternatively, antioxidant reserves may have been insufficient to counterbalance increased oxidant production, resulting in persistent oxidative burden despite enzymatic activation (Celi 2010; Lykkesfeldt and Svendsen 2007). These findings emphasize the importance of integrated oxidative stress indices such as OSI for assessing systemic redox status (Abuelo et al. 2013; Erel 2004, 2005).

The metabolic alterations observed in goats with kid mortality at birth further support the presence of impaired metabolic adaptation. The lower glucose concentrations detected in the kid mortality group suggest a more severe negative energy balance, a common feature of late gestation when energy demands exceed dietary intake (Bernabucci et al. 2005; Rook 2000). Similar metabolic changes have been reported in dairy goats during the transition period, characterized by substantial fluctuations in circulating metabolites associated with energy metabolism and physiological adaptation (Eşki et al. 2015; Soares et al. 2018). Reduced glucose availability may limit fetal energy supply and compromise neonatal viability, particularly during the critical transition to extrauterine life (Bell 1995; Rook 2000). In the present study, the higher total protein concentrations observed in goats experiencing kid mortality at birth may reflect inflammatory responses, dehydration-related hemoconcentration, or altered hepatic protein metabolism associated with metabolic stress (Celi 2010; Sordillo and Aitken 2009). Metabolic disturbances associated with negative energy balance and pregnancy-related disorders, such as pregnancy toxemia, have also been linked to alterations in oxidative status and systemic metabolic imbalance in small ruminants (Mohammed et al. 2023; Uztimür and Ünal 2024). These metabolic disturbances may exacerbate oxidative imbalance by increasing substrate oxidation and reactive oxygen species production, thereby creating a vicious cycle between metabolic strain and oxidative damage (Celi and Gabai 2015).

In the present study, unexpectedly, ALT activity was significantly lower in goats with kid mortality. Although elevations in liver enzymes are commonly associated with hepatocellular injury, reduced ALT activity may reflect altered hepatic metabolic capacity or adaptive metabolic responses during the periparturient period rather than direct liver damage. The transition period is characterized by profound metabolic adjustments in liver function related to energy balance, gluconeogenesis, and protein metabolism, which may influence circulating enzyme activities without necessarily indicating pathological changes (Radin et al. 2015; Soares et al. 2018). Previous studies in dairy goats have reported dynamic alterations in biochemical profiles during the periparturient period, reflecting physiological adaptations to increased metabolic demands and negative energy balance (Radin et al. 2015). Therefore, the lower ALT activity observed in the present study may indicate altered hepatic metabolic responses associated with impaired energy metabolism and systemic physiological stress in goats experiencing kid mortality.

The significant effects of time on most oxidative and biochemical parameters indicate that the periparturient period is characterized by dynamic metabolic and redox adaptations. These temporal changes are consistent with previous reports demonstrating substantial fluctuations in oxidative balance and metabolic profile from late gestation to early lactation (Bernabucci et al. 2005; Castillo et al. 2006; Celi 2010). The group × time interactions observed for several parameters suggest that goats experiencing kid mortality at birth may have different physiological adaptation patterns during the periparturient period. However, given the limited sample size and the absence of predictive performance analyses, these findings should be interpreted as exploratory associations rather than evidence of diagnostic or predictive utility. Further prospective studies with larger cohorts are required to determine whether oxidative stress markers such as TOS and OSI have practical value for monitoring periparturient health or predicting kid survival outcomes in dairy goats.

A limitation of the present study is the relatively small sample size, which reflects the low incidence of kid mortality at birth under controlled farm conditions. Although post hoc power analysis indicated adequate power for detecting large effects in primary oxidative stress markers, further studies with larger populations are required to confirm these findings and improve generalizability.

## Conclusion

In conclusion, the present study suggests that kid mortality at birth is associated with systemic oxidative imbalance and alterations in selected metabolic parameters during the periparturient period in higher-parity Saanen goats. Elevated TOS and OSI levels, together with changes in selected metabolic parameters, may reflect altered physiological adaptation in goats experiencing kid mortality at birth. However, due to the small sample size, post-parturition group allocation, and lack of predictive performance analyses, these findings should be interpreted as preliminary associations rather than evidence of causality or predictive applicability. Further large-scale prospective studies are needed to confirm these associations, clarify the underlying biological mechanisms, and determine whether oxidative and metabolic parameters have clinical or herd-level relevance for kid survival.

## Data Availability

Further information on the data and methodologies will be made available by the author for correspondence, as requested.
